# Oral health awareness and hygiene practices among Pakistani children: a cross-sectional survey

**DOI:** 10.3389/froh.2025.1709750

**Published:** 2026-01-12

**Authors:** Kanza Ahmed Chandio, Aminah Ikram Ullah, Muhammad Farrukh, Muhammad Anas, Yumnah Zubair, Jaber Hamad Jaber Amin

**Affiliations:** 1 Ziauddin College of Dentistry, Karachi, Pakistan; 2 Institute of Dentistry, CMH Lahore Medical College, Lahore, Pakistan; 3 Margalla Institute of Health Sciences, Rawalpindi, Pakistan; 4 Bacha Khan College of Dentistry Mardan, Mardan, Pakistan; 5 Khyber Medical University, Peshawar, Pakistan; 6 Karachi Medical and Dental College, Karachi, Pakistan; 7 Department of Neurosurgery, Atbara Teaching Hospital, Atbara, Sudan; 8 MBBS, University of Sinnar, Sinnar, Sudan

**Keywords:** child health, dental hygiene, oral health, Pakistan, pediatric dentistry

## Abstract

**Background:**

Maintaining optimal dental health during childhood is foundational for quality of life and prevention of common oral diseases, notably dental caries and periodontal conditions. Optimal pediatric oral health is essential for lifelong wellbeing, yet oral diseases remain prevalent among children globally.

**Objective:**

This study evaluates oral health awareness and hygiene practices among children in three major cities of Pakistan, aiming to highlight knowledge gaps and behavioral patterns.

**Method:**

A cross-sectional survey was conducted among 200 children aged 6–15 years from Karachi, Lahore, and Rawalpindi using a structured, pre-validated questionnaire. Consent was obtained from guardians, and children were assisted in the local language. Data were analyzed using SPSS (version 27).

**Results:**

Most participants were aware that brushing prevents dental problems and that excessive sugar consumption is harmful. While 61% believed twice-daily brushing was ideal, only 52% practiced it. Dental visits were primarily problem-driven; only 10.5% visited biannually. Non-recommended habits, such as nail biting, were common. Oral health information predominantly came from parents rather than schools.

**Conclusion:**

Despite adequate awareness, gaps exist between knowledge and practice in pediatric oral hygiene. School-based programs, improved parental education, and regular preventive dental visits are crucial to reduce oral disease burden in Pakistani children.

## Introduction

Maintaining optimal dental health during childhood is foundational for quality of life and prevention of common oral diseases, notably dental caries and periodontal conditions (1, 2). Early development of good oral hygiene habits is vital. Paedodontists play an essential role, addressing dental growth, identifying anomalies, and managing pediatric patients' behavioral responses (3). Problems such as dental caries are highly prevalent, especially in lower socioeconomic groups (4, 5), with notable implications on general health (6).

Developing countries like Pakistan face high rates of oral disease due to poor access to care and insufficient education (7). Rural populations have worse outcomes due to systemic inequities (8). Dietary shifts toward processed, sugary foods affect caries prevalence globally, including Pakistan's urban regions (9, 10). Early childhood dental neglect is common due to parental misconceptions about primary teeth (11, 12). The World Health Organization advocates for early oral health education and school programs, which have demonstrated efficacy internationally (13, 14).

Paedodontics assists in addressing challenges during a child's developmental phase by establishing and maintaining good dental care habits essential for preventing future oral health problems. These specialized dentists play a critical role in identifying early tooth decay, monitoring abnormalities in the growth of teeth and jaws, and managing the behavioral aspects of children during dental visits to reduce fear and enhance comfort (3). Oral diseases, including dental caries, are widespread yet frequently neglected, particularly among children from low-income families, minority groups, or those with limited access to dental care (1, 2). This neglect is alarming given the strong association between oral and general health; untreated cavities rank among the most prevalent pediatric conditions globally (4).

Developing countries such as Pakistan, India, and Bangladesh report high incidences of dental caries, periodontitis, and other oral diseases, primarily due to poor oral hygiene practices and low awareness levels (2, 5). In Pakistan, key factors contributing to this problem include inadequate access to dental services, insufficient routine check-ups, and gaps in health education (6–8). Moreover, recent evidence highlights that rural populations in Pakistan and India disproportionately suffer from poor oral health outcomes owing to systemic inequalities and limited public health investments (6, 8). Changing lifestyles have increased children's consumption of processed and sugary foods, a predominant cause of dental caries. For example, in the United Kingdom, nine out of ten children exceed recommended daily sugar intake (9). Similarly, Pakistan's urban centers face rising exposure to processed food marketing and availability (10). Lack of adequate dental care knowledge and awareness further exacerbates the risk of dental diseases in children (15).

Paedodontists emphasize the crucial role of parental guidance in maintaining children's oral health, advocating for early dental visits and regular check-ups (3). However, many parents remain unconcerned when their child's deciduous (primary) teeth develop caries, mistakenly believing these teeth will simply be replaced and do not require care. This misconception delays treatment and allows infections from primary teeth to adversely affect permanent dentition (12, 14). Studies corroborate low parental risk perception associated with primary teeth decay, underscoring the need for enhanced education (15).

Unfortunately, many children grow up without proper oral hygiene guidance from parents or schools. The World Health Organization advocates for oral health awareness from early childhood, including the promotion of regular tooth brushing to prevent diseases. School-based oral health programs have demonstrated success in improving knowledge and reducing plaque scores across diverse countries such as Thailand and Brazil (14, 16). Early prevention and treatment of dental caries are imperative, as untreated decay can cause pain, disrupted sleep, altered eating habits, speech difficulties, poor weight gain, and impaired growth (17, 18). Furthermore, early childhood caries often predict future decay (9). Understanding how families value primary teeth is essential for promoting prevention, necessitating collaboration among healthcare providers, educators, and communities (14, 19, 20).

Local data on Pakistani children's oral health awareness remain scarce. This study assesses awareness, habits, and information sources in children aged 6–15 in three cities, hypothesizing a gap between awareness and practice. In Pakistan, local records on pediatric dental health awareness and practices are scarce. This study aims to assess oral health awareness in children aged 6–15 years across three major cities (Lahore, Karachi, Rawalpindi). We hypothesis that there is a significant gap between oral health awareness and actual oral hygiene practices among pediatric patients in Pakistan.

## Materials and methodology

### Study design and participants

A cross-sectional survey was conducted in Karachi, Lahore, and Rawalpindi, three major cities of Pakistan. A convenience sample of 200 healthy children aged 6–15 was recruited from dental clinics, dental hospitals, and community centers. Convenience sampling was employed. Although a convenience sample was used, the sample size of 200 was determined based on an anticipated 36% prevalence of adequate oral health awareness among children (21), with a 6.6% margin of error and 95% confidence level by using WHO formula. This aligns with sample size recommendations for cross-sectional pediatric oral health surveys in similar resource-limited settings. We acknowledged that convenience sampling carries inherent bias, and we have addressed this in the Limitations section as recommended.

Data collection was conducted through a validated structured questionnaire distributed in these settings. Prior to participation, informed consent was obtained from the parents or guardians of the children. The questionnaire was then explained to the children in the local language, and they were assisted in answering where needed, to ensure accurate understanding and response. Given the wide age range (6–15 years), the questionnaire was explained verbally in the local language, and children were provided minimal assistance only for comprehension not for influencing answers to avoid interviewer bias.

### Questionnaire development and validation

The questionnaire was developed following a comprehensive literature review (22, 23) and adapted to local context including questions on brushing frequency, dental visits, sugary food effects, and oral health behaviors. Some questions were adapted from previous studies, such as how regularly did the child clean their teeth and visit their dentist (22, 23). Some were also adapted from the validated questionnaire used by Al-Omiri et al. (22) including those assessing brushing frequency, timing, dental visit habits, and awareness of caries and sugar-related oral health effects. To ensure validity, the modified questionnaire was further assessed through a pilot study involving 20 participants, with reliability confirmed via Cronbach's alpha analysis having a score of 0.78. These submissions were excluded from the main study.

The final survey included 19 items that were designed to evaluate participants' demographic characteristics, oral health knowledge, attitudes and behaviors. The format for the response included; writing the answer, selecting one option from a list and choosing more than one answer for the same question (Supplementary File 1).

### Ethics and data collection

A cross-sectional survey was conducted in Karachi, Lahore, and Rawalpindi with Institutional Review Board approval from Bacha Khan Medical College (15/02/2025) and study adhered to the Declaration of Helsinki. Consent was obtained from parents or guardians. The questionnaire was explained in the local language to children, who completed it alone with minimal assistance to avoid biasing responses. Only non-identifiable data were analyzed for confidentiality. After consent was secured, the questionnaire was explained to the children in the local language to ensure comprehension. The children then completed the questionnaire under supervision, with assistance provided for clarification where necessary. The authors did not directly assist in answering the questions to avoid influencing the responses.

Ethical considerations for this study include collecting participants' names solely for the purpose of consent verification; all identifying information was omitted during analysis to ensure data anonymity. The inclusion criteria comprised children aged 6–15 years who were healthy and free from severe systemic illnesses. The exclusion criteria included: children with special healthcare needs, those who did not fall within the specified age range, and those who declined to provide consent.

### Statistical analysis

Data were analyzed using SPSS v27 with descriptive statistics. Frequencies and percentages summarized awareness, habits, and attitudes.

## Results

A total of 200 pediatric dental patients (ages 6–15) participated, predominantly from Karachi (50%), followed by Lahore (30%) and Rawalpindi (20%). Males constituted 55% and females 45% of the cohort. The majority of children reported visiting a dentist only when experiencing pain or dental problems (59.5%), with just 10.5% attending regular biannual check-ups, and 11.5% visiting annually. Notably, 18.5% had never visited a dentist (Table 1).

**Table 1:** Frequency of dental visits among pediatric dental patients.

Frequency of dental visits	Percentage (%)
Only with pain/problems	59.5
Once a year	11.5
Every 6 months (routine)	10.5
Never visited dentist	18.5

This table summarizes the distribution of dental visit frequency among pediatric patients. The highest proportion (59.5%) reported visiting the dentist only when experiencing pain or dental problems. Other visit frequencies include annual visits (11.5%), routine biannual check-ups every six months (10.5%), and those who have never visited a dentist (18.5%). The data reflects utilization patterns of dental care in the studied pediatric population.

**Figure 1:**
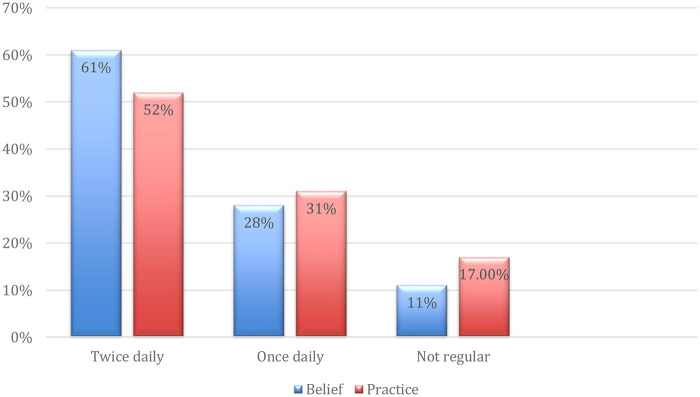
This bar chart illustrates the gap between participants’ beliefs about ideal brushing frequency and their actual behavior. While most believe in brushing twice a day, a lower percentage adheres to this practice, highlighting a persistent knowledge-practice gap.

A significant majority of the participants (85.5%) recognized the importance of brushing their teeth, and a similar proportion (84%) reported that they had received proper instructions on brushing technique. Despite this relatively high level of awareness and formal guidance, the knowledge did not consistently translate into optimal oral hygiene practices among the children. Additionally, 78.5% of the respondents were aware that sugary foods are harmful to dental health, while just over half (56.5%) demonstrated familiarity with the concept of dental cavities. These findings indicate that, although foundational knowledge of oral health is widespread, there remain gaps in comprehensive understanding and effective application of this knowledge in daily habits. While 61% believed brushing twice daily was ideal, only 52% actually practiced it. A notable number (8.5%) brushed more than twice, whereas another 8.5% did not brush regularly (Figure 1).

The majority of participants identified brushing their teeth after waking up in the morning as the best time to brush (71.5%), followed by before bedtime (49%) and after meals (44%). A small portion (7.5%) were unsure about the optimal timing. These responses indicate a generally good, though not complete, awareness of recommended tooth brushing times among the children surveyed. Almost four out of five children (78.5%) knew that sugary foods are detrimental, but only 21.1% reported rarely consuming sugary snacks or drinks. Most consumed such items once (40.2%) or more than once (38.7%) daily (Table 2).

**Table 2:** Frequency of sugary snack and drink consumption among participants.

Frequency of sugary snack/drink consumption	Percentage (%)
Once a day	40.2
More than once a day	38.7
Rarely	21.1

This table displays the percentage distribution of participants based on how often they consume sugary snacks and drinks. The majority reported consuming these items once a day (40.2%) or more than once a day (38.7%), while a smaller proportion (21.1%) reported rarely consuming sugary snacks or drinks. The data reflects dietary behavior relevant to oral and general health patterns in the studied population.

When asked which factors could harm teeth, 78.5% of children highlighted sugary snacks, 51.5% soft drinks, and 39.5% chewing gums with sugar. Surprisingly, 4% mistakenly regarded regular brushing as harmful, pointing to persistent misinformation. A substantial proportion of the children surveyed reported engaging in potentially harmful oral habits (Table 3). The findings underscore the primary role of parents and dental professionals as sources of oral health education, while revealing an opportunity for greater involvement from schools and digital media platforms. This distribution highlights the crucial role of parents and dental professionals in imparting oral health education, while also indicating potential for increased educational input from schools and digital media.

**Table 3:** Survey findings on children's oral health habits, beliefs, and information sources.

Category & findings	Percentage (%)
A. Engagement in potentially harmful oral habits	
Frequently (e.g., open packages, bite nails, chew pencils)	42.5
Sometimes	32.5
Never	25.5
B. Belief regarding dental aesthetics	
Believe white/straight teeth are important for a good smile	80.0
C. Primary source of oral health information	
Parents	41.0
Dentists	32.0
Schools	19.5
TV or Internet	7.5

Key Interpretation: A substantial majority (75.5%) of children reported engaging in harmful oral habits to some degree, indicating prevalent para-functional activities with potential negative impacts on dental health and development.

## Discussion

This research provides a detailed summary of children aged 6–15 years in Pakistan, focusing on their understanding, awareness, and habits related to dental care in Pakistan, highlighting several areas needing improvement. The results confirm that, although most children have basic awareness, gaps persist in preventive behavior, knowledge dissemination, and frequency of dental visits.

The findings reveal reasonable oral health knowledge yet discrepancies between knowledge and behavior, consistent with prior regional studies (5, 15). Notably, only half brush twice daily despite 61% endorsing that frequency, indicating a knowledge-practice gap. Non-recommended para functional habits such as nail biting appeared prevalent, which corresponds with increased risk of malocclusion and enamel wear (19, 20). Parents were the leading source of oral health information, highlighting the need to educate caregivers comprehensively (12). The low engagement from schools suggests missed opportunities for early education, as school-based programs have proven effective globally (11). Regular dental visits remain a challenge; only 10.5% attended preventive checkups, matching patterns seen in South Asia and the Middle East (24, 25). Promoting early dental visits and prioritizing preventive care are key steps toward transforming the predominantly reactive dental care.

This study pointed towards some alarming findings, such as 43% of children engaging in behaviors like biting their nails or opening packages with their teeth, habits associated with enamel damage. Pediatric dentists agree that such habits, especially if they persist beyond ages 3–4, are harmful (20). Yet only 35% of participants reported consistently following dental advice, underscoring challenges in behavioral compliance. Parents and teachers must monitor and guide children to help eliminate such habits, as they are risk factors for malocclusion (20). Studies show that pediatric patients with these habits have a high incidence of malocclusion (20). This is also emphasized in findings from South Asian cohorts, where para functional habits were correlated with anterior open bite and cross bite (19). Moreover, 80% of children believed that having white or straight teeth is essential for a good smile, reflecting growing aesthetic awareness. This could indicate a shift in perception where appearance is beginning to motivate oral care. Increased influence of social media on children's perceptions of dental aesthetics has also been reported in recent years (26).

Only 19.5% of children reported learning about oral health from school, suggesting a missed opportunity for school-based oral health education. Some private schools in major cities have included basic oral hygiene modules in health science curricula, but no widespread implementation exists [14. Studies from Brazil and Indonesia confirm that consistent school-based oral health programs can greatly lower the percentage of cavities in children (11, 27). Although 84% had received brushing instructions and 85.5% knew about the need to brush, only 52% actually brushed twice a day indicating a knowledge-behavior gap. This discrepancy, seen in studies like Schroth, Brothwell, and Moffatt (2007) (15), is influenced by parental beliefs and cultural values. For example, when caregivers devalue primary teeth, believing they will fall out anyway, children adopt poor hygiene habits, leading to higher early childhood caries (14). Bridging this gap requires educating not only children but also their caregivers.

Knowledge about dietary risks was also fragmented. While 78.5% knew that sugary foods harm teeth, only 39.5% were aware that sugary chewing gum is harmful, and 4% incorrectly believed brushing damages teeth. These misconceptions likely stem from non-dental sources like peers, media, or uninformed parents. Since 41% cited parents as their main information source and only 32% mentioned dentists, these findings reflect caregiver influence, consistent with earlier research (14). This is reinforced by findings in rural India, where media misinformation about oral health was common and parental knowledge often outdated (28).

Our study aligns with international findings (5, 29, 30). For instance, a study in rural Bangladesh revealed that most students visited dentists only in emergencies (5). Similarly, in our study, only 10.5% of children reported visiting the dentist twice a year as recommended, while 59.5% visited only during pain or issues. The American Academy of Pediatrics suggests that a child should have their first visit to the dentist by the age of one, followed by routine checkups every six months (9, 14, 18). Yet, as noted by Bhaskar et al. (2014) and Ozveren et al. (2021) (14, 25), this is rarely followed in developing regions. Recent reports from the Middle East echo these trends, attributing delays to cost, fear, and cultural barriers (24). Our research supports the conclusion that despite global shifts toward preventive care and caries risk classification (18), dental care in Pakistan remains largely reactive. Treatment is prioritized over prevention, and structured oral health strategies are largely missing. Furthermore, national-level surveillance and oral health promotion policies are insufficiently implemented in Pakistan, limiting the scalability of successful interventions (31, 32).

It is imperative to introduce coordinated national dental health campaigns aimed at school-age children. Introducing oral health modules in school curricula, dentist-led outreach in underserved areas, and improved parental education can cultivate a more informed pediatric population (33, 34). Early prevention is critical to avoid long-term consequences of childhood caries, such as speech issues, nutritional problems, and psychological impacts (2, 7, 9). Similar national programs in different countries have demonstrated strong long-term oral health outcomes (16, 35). This study has several limitations, including an urban-focused sample and the possibility of misunderstanding among younger participants despite pilot validation an issue commonly observed in pediatric survey research (23, 28). First, although the questionnaire was explained to children in the local language, younger participants may still have misunderstood some questions, potentially introducing response bias. Second, convenience sampling reduces the generalizability of the results to the larger Pakistani paediatric community. Thirdly, the survey did not address other behaviors, like thumb sucking, lip biting, mouth breathing, and tongue thrusting. Furthermore, the urban bias, especially the great representation of Karachi, may not entirely reflect oral health practices in rural areas. To present a more complete picture, future studies including bigger, randomized, geographically scattered samples are required.

## Conclusion

Pakistani children know the basics of oral health but frequently fail to practice adequate hygiene and prevention. Coordinated efforts involving schools, parents, and healthcare providers are needed to promote preventive care and regular dental visits. This study lays groundwork for targeted interventions to improve pediatric oral health outcomes in Pakistan. This study also provides a basis for next treatments meant to enhance the oral health results among Pakistani children.

Importantly, this study contributes new evidence by simultaneously evaluating oral hygiene practices, harmful habits, dietary patterns, and information sources among children from three major urban centers. It highlights how parental influence, limited school-based education, and inconsistent preventive dental visits collectively shape children's oral health behaviors. By identifying these multi-level determinants, this research provides a much-needed foundation for designing targeted public health strategies, especially in a context where national pediatric oral health data remain scarce.

Moving forward, future studies should include larger and more geographically diverse samples, incorporate rural populations, and explore parental attitudes, school-based factors, and behavioral determinants in greater depth. National preventive programs, integration of structured oral health modules in school curricula, and stronger parent-centered education initiatives are essential to bridge the knowledge practice gap. Strengthening collaboration between parents, schools, and dental healthcare providers will be critical to improving long-term oral health outcomes among Pakistani children.

## Data Availability

The original contributions presented in the study are included in the article/Supplementary Material, further inquiries can be directed to the corresponding author.
