# Supplementary material for: Oral health awareness and hygiene practices among Pakistani children: a cross-sectional survey
Source: Front Oral Health. 2026 Jan 12;6:1709750. doi: 10.3389/froh.2025.1709750 (PMC12832794; doi:10.3389/froh.2025.1709750)
Supplement: Supplementary file 1 [file Datasheet1.docx]

**ORAL HEALTH AWARENESS & HYGIENE PRACTICES QUESTIONNAIRE**

**Informed Consent and Introduction**

**Instructions:** Dear Participant/Parents,
You are invited to take part in a research study. Your participation is entirely voluntary and anonymous. Your responses will be kept confidential and used solely for academic research purposes. Completing this survey implies that you have read this information and provide your informed consent to participate. The survey should take approximately 5-7 minutes to complete.

***For Children Aged 6–15 Years***

**Instructions for Children:**
• This questionnaire is about your tooth-cleaning habits and oral health knowledge.
• There are no right or wrong answers, please answer honestly.
• You may ask for help in reading or understanding a question.
• Do not let anyone choose the answer for you.

**Section A: Demographic Information**

1. **What is your age?**
   ☐ 6–8   ☐ 9–11   ☐ 12–13   ☐ 14–15
2. **What is your gender?**
   ☐ Male   ☐ Female
3. **Which city do you live in?**
   ☐ Karachi   ☐ Lahore   ☐ Rawalpindi Other
4. **What type of school do you attend?**
   ☐ Government/Public
   ☐ Private
   ☐ Not in school
5. **Highest education level of your parents (optional):**
   **Father:** ☐ Primary ☐ Secondary ☐ College+
   **Mother:** ☐ Primary ☐ Secondary ☐ College+

**Section B: Oral Hygiene Knowledge**

1. **Do you think brushing your teeth is important?**
   ☐ Yes   ☐ No   ☐ I don’t know
2. **How often do you think people should brush their teeth?**
   ☐ Once a day
   ☐ Twice a day
   ☐ More than twice a day
   ☐ I don’t know
3. **Which one can cause tooth decay? (Select all that apply)**
   ☐ Eating sugary snacks
   ☐ Drinking soft drinks
   ☐ Chewing sugary gum
   ☐ Not brushing teeth
   ☐ Regular brushing harms teeth (Incorrect but included to assess misconceptions)
   ☐ I don’t know
4. **Have you learned how to brush your teeth properly?**
   ☐ Yes
   ☐ No
   ☐ Not sure

**Section C: Oral Hygiene Practices**

1. **How often do you actually brush your teeth?**
   ☐ Once a day
   ☐ Twice a day
   ☐ More than twice a day
   ☐ Not every day
2. **When do you usually brush your teeth? (Select all that apply)**
   ☐ After waking up
   ☐ Before going to bed
   ☐ After meals
   ☐ I forget / don’t brush regularly
3. **How long do you brush your teeth each time?**
   ☐ Less than 1 minute
   ☐ 1–2 minutes
   ☐ More than 2 minutes
   ☐ I don’t know

**Section D: Dietary Practices**

1. **How often do you eat sugary snacks or drinks?**
   ☐ Rarely
   ☐ Once a day
   ☐ More than once a day
2. **Which sugary items do you frequently consume? (Select all that apply)**
   ☐ Chocolates
   ☐ Biscuits/cakes
   ☐ Soft drinks
   ☐ Sweet juices
   ☐ Sweet chewing gum
   ☐ None

**Section E: Dental Visit Behavior**

1. **How often do you visit the dentist?**
   ☐ Only when I have pain/problem
   ☐ Once a year
   ☐ Every 6 months (routine)
   ☐ I have never visited a dentist
2. **Why did you last visit the dentist?**
   ☐ Pain
   ☐ Cavity
   ☐ Routine check-up
   ☐ Cleaning
   ☐ Never visited

**Section F: Harmful Oral Habits**

1. **Do you have any of these habits? (Select all that apply)**
   ☐ Bite nails
   ☐ Chew pencil/pen
   ☐ Use teeth to open things
   ☐ Thumb sucking / lip biting
   ☐ None
2. **How often do you do these habits?**
   ☐ Frequently
   ☐ Sometimes
   ☐ Never

**Section G: Information Sources**

1. **Where did you learn most about taking care of your teeth?**
   ☐ Parents
   ☐ Dentist
   ☐ School
   ☐ TV / Internet / Social Media
   ☐ Friends
   ☐ No one taught me

**End of Questionnaire**

✓ Thank you for participating!
✓ Your answers will help improve children’s dental health programs.
